# Magnetic Trapping of Bacteria at Low Magnetic Fields

**DOI:** 10.1038/srep26945

**Published:** 2016-06-02

**Authors:** Z. M. Wang, R. G. Wu, Z. P. Wang, R. V. Ramanujan

**Affiliations:** 1 School of Materials Science and Engineering, Nanyang Technological University 50 Nanyang Avenue, Singapore 639798, Singapore; 2 Singapore Institute of Manufacturing Technology, 71 Nanyang Drive, Singapore 638075, Singapore

## Abstract

A suspension of non-magnetic entities in a ferrofluid is referred to as an inverse ferrofluid. Current research to trap non-magnetic entities in an inverse ferrofluid focuses on using large permanent magnets to generate high magnetic field gradients, which seriously limits Lab-on-a-Chip applications. On the other hand, in this work, trapping of non-magnetic entities, e.g., bacteria in a *uniform* external magnetic field was studied with a novel chip design. An inverse ferrofluid flows in a channel and a non-magnetic island is placed in the middle of this channel. The magnetic field was distorted by this island due to the magnetic susceptibility difference between this island and the surrounding ferrofluid, resulting in magnetic forces applied on the non-magnetic entities. Both the ferromagnetic particles and the non-magnetic entities, e.g., bacteria were attracted towards the island, and subsequently accumulate in different regions. The alignment of the ferrimagnetic particles and optical transparency of the ferrofluid was greatly enhanced by the bacteria at low applied magnetic fields. This work is applicable to lab-on-a-chip based detection and trapping of non-magnetic entities bacteria and cells.

Ferrofluids are stable colloidal dispersions of surfactant coated ferrimagnetic nanoparticles (MNPs, usually with particle diameter of ~10 nm or less) in a carrier fluid, e.g., water or organic oil^1^. Besides these MNPs, non-magnetic entities (with negligible magnetic susceptibilities), e.g., biological entities, can be dispersed in the ferrofluid, this mixture is called an inverse ferrofluid^2^. Current research on inverse ferrofluids mainly focuses on its rheological properties and particle separation using non –uniform magnetic fields.

Conventionally, non-magnetic entities within the fluid can be detected or trapped by an external magnetic field *gradient*, based on the magnetophoresis principle that particles are directed either along or against the magnetic field gradient^3^. Such magnetic field-induced particle manipulation is used for microfluidic Lab-On-a-Chip applications as it is simple, cheap, and free of undesirable fluid heating that accompanies electric, acoustic, and optical methods^3^. Two methods are commonly used a): the target non-magnetic entities are chemically attached to MNPs which are suspended in a non-magnetic carrier fluid like water. The MNP-non-magnetic entity complex experiences positive magnetophoresis^4^, and would be attracted towards the magnetic field source^5^,^6^,^7^,^8^,^9^. This method is used to selectively trap and continuously sort cells or biomolecules from a heterogeneous mixture by labeling target biological entities with functionalized MNPs^10^,^11^,^12^,^13^. However this method is of limited practical significance due to the need for complex procedures to bind the MNPs to the non-magnetic entities. b) the non-magnetic entities in an inverse ferrofluid can be concentrated by negative magnetophoresis since they would be repelled from the source of the magnetic field^3^,^14^,^15^. The attraction of the MNP by the external field gradient will generate a fluid pressure gradient which will levitate the non-magnetic entities in the opposite direction^14^,^16^. This force can be called the magnetic buoyancy force. The separation efficiency of non-magnetic entities usually depends on the relative position of the particle and the magnetic field source, strength of the magnetic field gradient, particle size, ferrofluid concentration, susceptibilities, and flow rate^3^.

However, both these methods require high magnetic forces; usually one^17^,^18^ or a few^15^,^19^,^20^,^21^ permanent magnets have to be placed adjacent to the channel in which fluid flow is occurring in order to generate a non-uniform magnetic field. Since the fields of micro magnets are usually insufficient to generate a high magnetic field gradient, the sizes of the magnets are much larger than the channel, which is usually a few μm wide. Also, the efficiency of separation depends to a great extent on the geometry of the magnet and its position. To obtain large magnetic field gradients, the magnets are usually closely placed near the channel, a small displacement of the magnet’s position can significantly influence the field gradient. Small electromagnets^22^ can generate *non-uniform* magnetic fields, but suffer similar limitations. Hence, the use of *non-uniform* magnetic fields seriously limits magnetic Lab-On-A-Chip applications.

For example, a linear array of rectangular soft-magnetic elements was embedded beneath a microfluidic channel and magnetized using an external bias field^23^,^24^,^25^, and used to capture magnetic particles in a bioseparation microsystem but this method is complex.

Previous theories on particle manipulation driven by magnetic forces mainly focus on the magnetization of magnetic particles which can cause dipolar interactions between magnetic particles^26^,^27^,^28^,^29^,^30^,^31^,^32^. Less attention has been paid to physical interactions between non-magnetic and ferromagnetic entities.

On the other hand, we previously reported^33^ that an external uniform magnetic field could be *locally* distorted by mixing entities that have different magnetic susceptibilities compared to that of the carrier fluid, this distortion generated a gradient of magnetic field, leading to magnetic forces. Thus, in this work, a design is proposed with a simple *uniform* external magnetic field making magnetic field induced particle manipulation a compact, “wireless”, technique. A rectangular channel was milled away from a chip of PMMA, except for an elliptical pillar “island” remaining, in the middle of this channel (Fig. 1). When the channel was filled with an inverse ferrofluid containing non-magnetic entities (e.g., bacteria with negligible magnetic susceptibility), this island would be surrounded by this fluid. When a uniform magnetic field was applied, it was found that the MNP and the non-magnetic entities would drift towards different regions of the island, resulting in separation and trapping. Enhancement of MNP alignment and fluid optical transparency was observed and investigated as a function of magnetic field.

## Results

### Alignment of Magnetic Particles Enhanced by Bacteria

**Figure 1:**
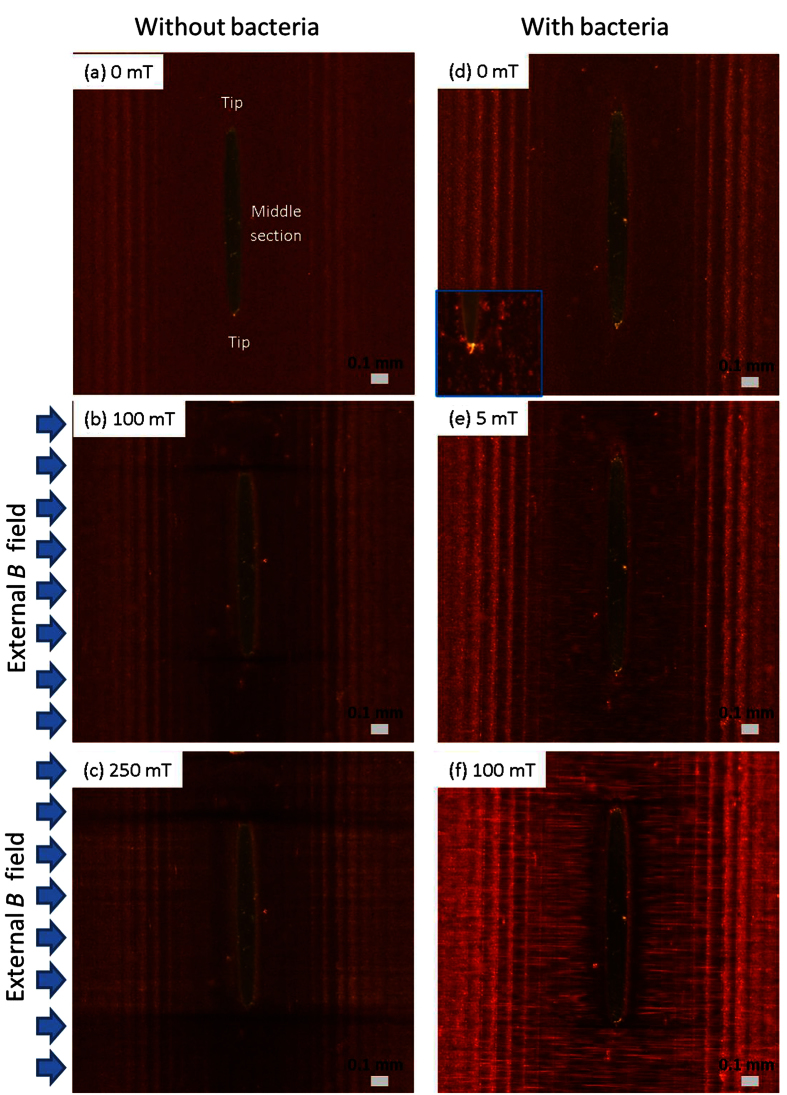
Alignment and distribution of MNPs in the ferrofluid (**a–c**) without, and (**d–f**) with bacteria, at an applied magnetic flux density *B* of (**a,d**) 0 mT, (**e**) 5 mT, (**b,f**) 100 mT and (**c**) 250 mT. The bright dots in inset of (**d**) indicate bacteria around the bottom tip.

In this work, the external uniform magnetic field direction was from left to right. Figure 1 shows the alignment of MNPs without (a–c) and with (d–f) bacteria for a range of magnetic fields, in regions far from the island. Figure 1a and the Fig. 1d shows the MNP distribution for zero magnetic field. The bright dots in inset of Fig. 1d were bacteria cells because they can only be observed when the ferrofluid was mixed with bacteria. At higher applied magnetic field, MNPs alignment was observed along the field direction in regions far from the island. For ferrofluid without bacteria and with an applied field of 100 mT (Fig. 1b), almost no alignment was observed in regions far away from the island. However, with a magnetic field of 250 mT (Fig. 1c), alignment was observed.

Interestingly, with bacteria mixed with the ferrofluid (Fig. 1e,f), needle shaped alignment could be observed in regions far from the island at much lower magnetic fields, e.g., at 5 mT. At 100 mT, long ferrimagnetic needles were observed.

### Magnetic and Non-magnetic Particles Rearrangement

Figure 1 also showed the distribution of MNPs in the ferrofluid mixed without (a–c) and with (d–f) bacteria. Without an applied magnetic field (0 mT), the MNPs in a ferrofluid, with or without bacteria, were uniformly distributed (Fig. 1a,d). When the magnetic field was applied (100 mT, Fig. 1b,f), besides the alignment of MNPs in regions *far from the island*, MNPs near the island were also attracted and concentrated near the top and bottom tips of the island to form needle shaped clusters, however, MNP are absent from the middle section of the island. Interestingly, similar results due to small bubbles instead of an island (figure not shown) were also observed, MNP alignment was observed near the top and bottom sections of the bubbles.

**Figure 2:**
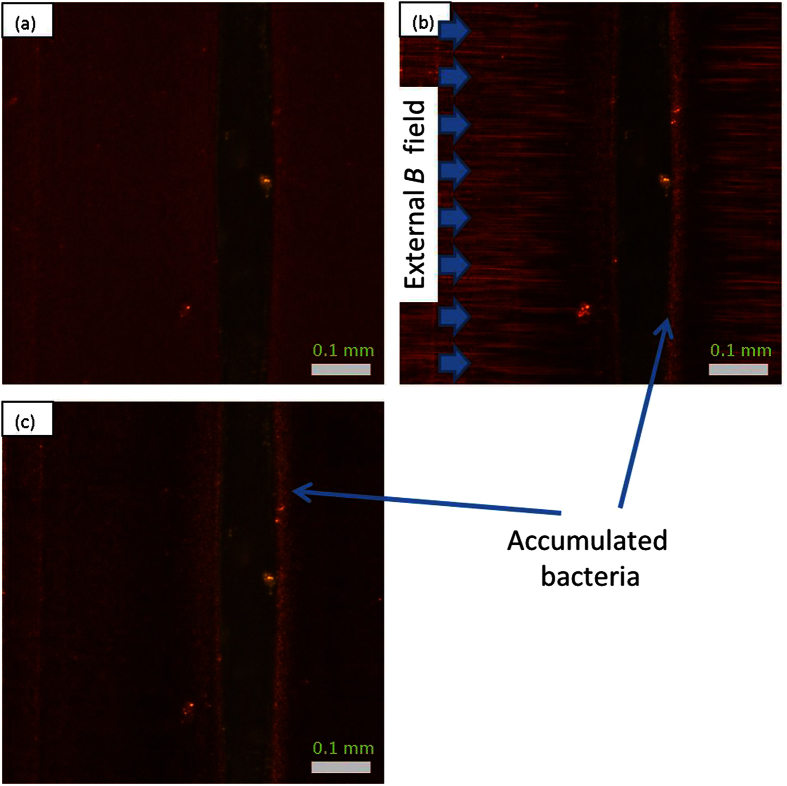
Magnified view of middle section of island, showing bacteria accumulation with *B* increased from (**a**) 0 mT to (**b**) 200 mT, and finally lowered back to (**c**) 0 mT.

In the presence of an applied magnetic field, the island could attract and accumulate magnetic particles as well as non-magnetic entities, e.g., bacteria. As shown in Fig. 2a, without applied magnetic field (0 mT), the bacteria were uniformly distributed in the ferrofluid. When magnetic field was increased to 200 mT, it was observed that bacteria (white dots) accumulated near the middle section of the island (Fig. 2b), and remained in this section (Fig. 2c), even after the magnetic field was reduced from 200 mT to zero.

From Figs 1 and 2, it was concluded that MNPs could be attracted to the tips of the island, and were absent from the middle sections. On the other hand, the non-magnetic particles accumulated at the middle section of the island.

### Optical Studies

**Figure 3:**
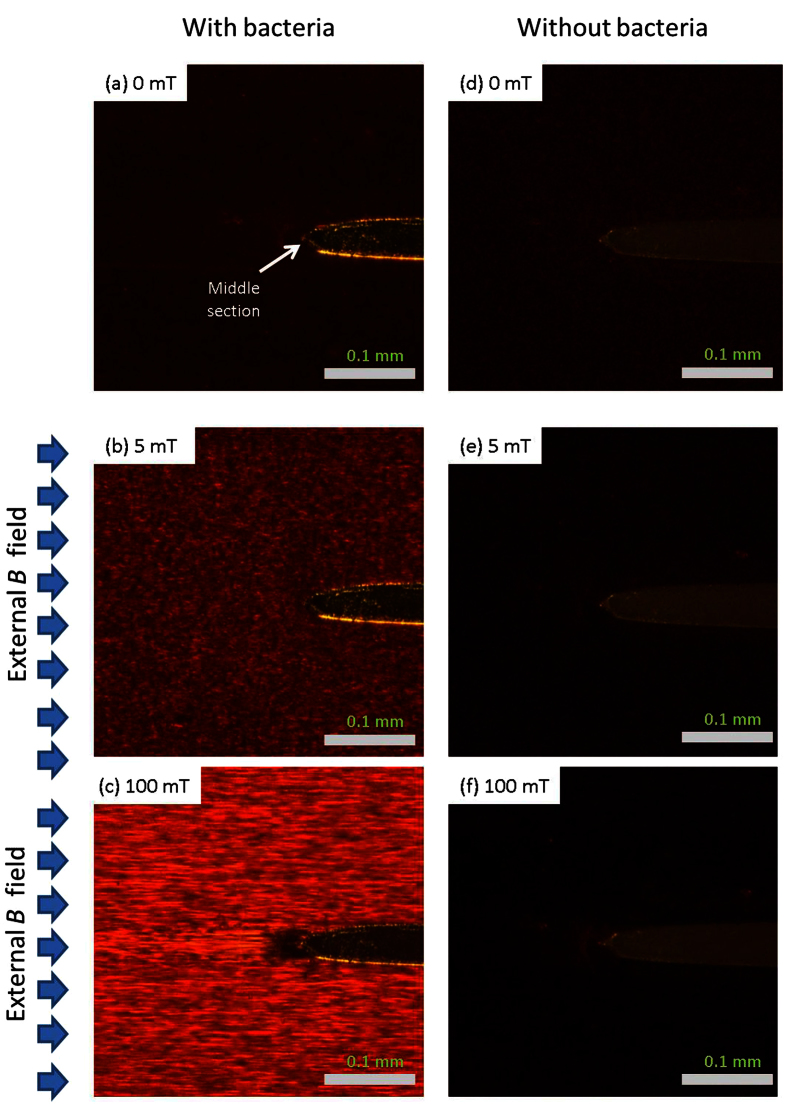
Optical study of channels filled with ferrofluid with or without bacteria, all with same light intensity. The major axis of the island is parallel to the field direction, from left to right.

For the optical transparency study, the channel was placed parallel to the magnetic field, so that the major axis of the island was parallel to the magnetic field direction (left to right in Fig. 3). The light source was placed directly below (vertically) the chip so that better color contrast could be observed compared with previous studies.

As shown in Fig. 3a,d, without applied magnetic field (0 mT), the ferrofluid mixed with bacteria was dark, while the transparent island was much brighter, indicating low transparency of ferrofluid without magnetic field.

With increasing magnetic field, for the ferrofluid mixed with bacteria, MNP alignment could be clearly observed in the regions further away from the island as shown in Fig. 3b,c, but much brighter than the previously discussed photos (Fig. 1e,f) due to the vertical placement of light source.

Compared with Fig. 3a, at applied magnetic field of 5 mT (Fig. 3b), the ferrofluid and bacteria mixture showed increased brightness, indicating that transmittance was increased due to MNP alignment. Transmittance could be further improved at magnetic field of 100 mT (Fig. 3c), with bright and dark needle shaped MNP alignments. For comparison, the optical properties of ferrofluid without bacteria only changed slightly when magnetic flux density increased from 0 to 100 mT, as shown in Fig. 3(d–f).

The average lightness ratio (*R*_*I*_) of ferrofluid over island could be used to represent the transmittance of ferrofluid compared with the island, where the lightness (*I*) could be calculated using a formula^34^ of *I* = (*R* + *G* + *B*)/3, where *R, G* and *B* are the RGB (red, green and blue) space values of randomly selected points in the photos. With magnetic field increased from 0 to 5 and 100 mT, the averaged *R*_*I*_ values over 30 measurements of each case for Fig. 3a–c (with bacteria) increased from 0.88 to 1.49 and 2.58, respectively (with standard deviations of 0.0322, 0.0278 and 0.0391, respectively), but not much change (0.47, 0.39 and 0.41, respectively, with standard deviations of 0.0446, 0.0427 and 0.0371, respectively) was observed for Fig. 3d–f (without bacteria).

## Discussion

### Modelling of Trapping

**Figure 4:**
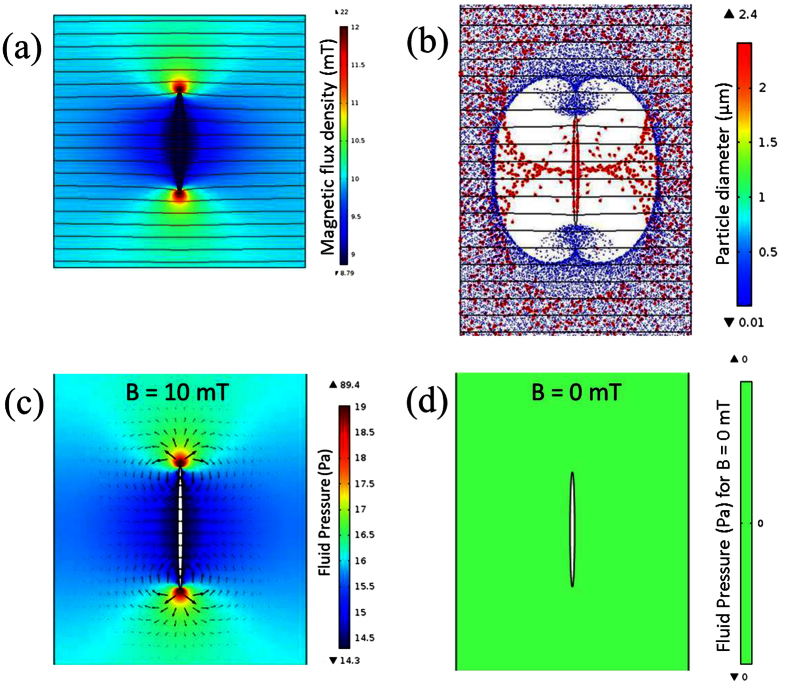
(**a**) Simulated distribution of magnetic flux density *B* [mT] and (**b**) shows the simulated distribution of MNPs (small dots) and bacteria (large dots) when a magnetic field of 10 mT was applied. Particle sizes are not to scale. The 2D contour plots of the *z* component of magnetic vector potential *Vz* [Wb/m] are also shown in (**a,b**) for an external uniform magnetic flux density of 10 mT, applied from left to right. Figure (**c,d**) shows the color plotting of simulated fluid pressure distribution due to the magnetic forces applied on ferrofluid with B = 10 mT and 0 mT, respectively, and the arrows in (**c**) show both the magnitudes (log format) and directions of corresponding magnetic buoyancy forces.

In a medium consisting of materials with different magnetic susceptibilities, the magnetic field lines would bend towards regions with higher magnetic susceptibilities, and away from non-magnetic materials. Figure 4a showed a 2D simulated map of magnetic flux density inside a channel filled with ferrofluid, with an elliptical plastic island in the middle of the channel. If there was no non-magnetic island in the channel, the magnetic susceptibility and magnetic field flux density inside the channel should be uniform and equal to that of the ferrofluid (1.51) and 10 mT, respectively.

Due to the negligible magnetic susceptibility of the island, the magnetic flux density *B*, was not uniform (Fig. 4a): *B* had its maximum values (~22 mT) near the top and bottom tips of the island, and minimum values (~ 8.8 mT) inside the island.

This non-uniform distribution of magnetic flux density could also be described by the contour plots of magnetic vector potential. The magnetic field lines were distorted by the island: denser near the two tips and less dense near the middle sections of the island, resulting in magnetic field gradients, hence there were magnetic forces on the MNPs.

For better understanding, another model was developed with the same applied external magnetic fields (10 mT, horizontal from left to right), but converting the relative susceptibilities (*χ*_*r*_ = *χ* + 1) of the ferrofluid and middle island from *χ*_*ff*_ + 1 = 1.51 + 1 = 2.51 and 0 + 1 = 1, respectively, to 1 and 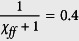, respectively, with the relative susceptibility ratios between the ferrofluid and the island maintained. If the real susceptibilities of the surrounding and island materials are *χ*_1_ and *χ*_2_, respectively, then the converted susceptibilities (*χ*_*con*_) are shown in Table 1.

In this case, the susceptibility of surrounding ferrofluid (*χ*_1_) and the middle non-magnetic island (*χ*_2_) are equal to 1.51 and 0, respectively, and the corresponding converted susceptibilities (*χ*_*con*_) will be 0 and −0.6, respectively. This is similar to the case where an elliptical island with susceptibility of −0.6 is placed in air. The simulated results of *B* and *Vz* (figure not shown) using the converted susceptibilities (*χ*_*con*_) are exactly the same as those in Fig. 4a. Thus the *B* field distributions depends on the relative susceptibility ratios of the two materials (e.g., 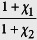), rather than the magnitude of the difference in susceptibilities.

Hence, the field distortion by a non-magnetic island surrounded by a ferrimagnetic material can be converted to the case of a material with negative susceptibility surrounded by air or other non-magnetic materials. Compared with the surrounding ferrimagnetic materials (e.g., ferrofluid) with higher susceptibility, the middle island (or empty region like a bubble) with lower susceptibility (*χ*_1_ > *χ*_2_) can be treated as a magnetic material with “relative-negative” susceptibility.

### Magnetic and Non-magnetic Particles Rearrangement

A particle tracing model was also used to numerically study the influence of island on the MNP and bacteria rearrangements (Fig. 4b). In the absence of applied magnetic field, MNPs were uniformly distributed inside the channel (except the island region where there were no MNPs). For a small external *uniform* magnetic flux density of 10 mT, the non-uniform distribution of magnetic flux density in Fig. 4a would lead to a gradient of the magnetic field, resulting in magnetic forces applied to the MNPs. The MNPs inside the ferrofluid would drift towards higher magnetic flux density regions (Fig. 4b), resulting in enrichment at the tips, which was same as described earlier in Fig. 1b,c.

For the inverse ferrofluid in this work, the diameter of the non-magnetic particles, e.g., bacteria (~ 2.4 μm), was typically several orders of magnitude larger than the nanosized MNPs (~10 nm). Hence, the ferrofluid (a mixture of carrier fluid and MNPs) could be treated as a hydrodynamically continuous and uniform fluid, and the bacteria could be treated as dispersed droplets. The magnetic forces were much greater on the magnetic ferrofluid compared to the non-magnetic particles, which generated a fluid pressure gradient inside the ferrofluid. The Fig. 4c,d shows the fluid pressure distribution of ferrofluid around the island for B = 10 mT and 0 mT, respectively. With B = 0 mT, the ferrofluid performs as a normal fluid like water, and since the pump was switched off during all measurements, the fluid pressure should be uniform and equal to 0 Pa (with a reference pressure of 1 atm) as shown in Fig. 4d. But with B = 10 mT, due to the magnetic forces applied on the ferrofluid the fluid pressure has its highest values near the two tips of island, and lowest values near the middle section of the island as shown in Fig. 4c. The variations of fluid pressure, which is the fluid pressure gradients, resulted in a “magnetic buoyancy” force on the bacteria (the arrows shown in Fig. 4c). This is similar to buoyancy forces on a rising bubble inside water due to the pressure gradient caused by gravitational force. Since the “magnetic buoyancy” force directions were opposite to the magnetic forces applied on the ferrofluid, the bacteria around the island accumulated near the middle sections (Fig. 2b,c). This could also been explained using the “relative-negative” susceptibility model as previously described in this work. The suspended non-magnetic particles (bacteria) had “relative-negative” susceptibility compared with the ferrofluid, so the bacteria experienced negative magnetophoresis, and were repelled from the high magnetic field regions (Fig. 4b, and a video of simulation was included as supplementary material). The experimental results in Fig. 2 confirmed this bacteria accumulation with applied magnetic field.

Thus, the efficiency of magnetic and non-magnetic particle trapping depends on the magnitude of the uniform applied magnetic field, relative susceptibility ratios of the two materials 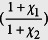, particle size, and appropriate channel design.

### Alignment of Magnetic Particles Enhanced by Bacteria

Compared with ferrofluid, the relatively higher magnetic susceptibilities of a MNP could bend the external initially uniform magnetic field lines towards the inside of this MNP, and this could generate magnetic field gradients around this MNP, leading to magnetic forces on the MNPs around this MNP. This would lead to the MNP to align in the direction of magnetic field to form MNP chains. This explanation was different from the previous theory using dipolar interactions between the MNPs (MNP-MNP interaction) caused by a magnetic field^26^,^27^,^28^,^29^,^30^.

Similarly as MNPs, using the “relative-negative” susceptibility model, the bacteria suspended in ferrofluid should also bend the magnetic field lines away from the inside of the bacteria, resulting in alignment to form bacteria chains (bacteria-bacteria interaction).

Due to the much larger size of a bacterium compared with MNPs, bacteria would also distort the magnetic fields in the ferrofluid, thus each bacteria cell can be treated as a micron sized non-magnetic island. Thus MNPs should prefer to accumulate near the top and bottom tips of the bacteria (similar to Fig. 4b). The definition of the positions of “the top and bottom tips” of the bacteria were similar to the one for the island inside the channel, the connecting line of those two tips was perpendicular to the magnetic field. The accumulated MNPs near the tips of the bacteria would form MNP clusters, resulting in increased local susceptibilities. Due to this MNP-bacteria interaction, a bacteria cell between two MNP clusters links those two clusters.

For the case of a non-magnetic island in the channel, the relatively higher MNP concentration in the clusters induced needle-shaped alignment of MNPs much more easily, even at much lower magnetic fields (Fig. 1b,c). Similarly, by treating a bacterium as a non-magnetic island, we can explain the reason that in the regions far away from the island, the MNP alignment can be enhanced at low magnetic flux density by mixing ferrofluid with bacteria (Figs 1e,f and 2b).

Two needle-shaped MNP clusters from two nearby bacteria would interact with each other, align and merge into a single, longer, needle-shaped MNP cluster, aligned along the magnetic field lines. These MNP-MNP interactions also align the bacteria that attach to those clusters.

Thus, the three kinds of particle interactions, i.e., MNP-MNP, bacteria-bacteria and MNP-bacteria were observed. Interestingly, this would lead to a net of bacteria and MNP clusters, with alternating kinds of chains: MNP-MNP alignment and bacterium-bacterium alignment, and these alignments can be cross-linked due to MNP-bacteria interactions.

### Optical Transmission

The dark brown MNPs dispersed inside the ferrofluid would reduce light transmission. When magnetic fields were applied to induce MNP alignment, most of the MNPs accumulated along the alignment lines, leaving the remaining regions with fewer MNPs. This “cleaned” ferrofluid greatly enhanced light transmission (Fig. 3). Thus transmission depended on the MNP alignment, and the presence of bacteria in the ferrofluid could enhance MNP alignment as previously discussed, hence the mixture of bacteria inside the ferrofluid resulted in enhancement of light transmission, even at low magnetic field (Fig. 3b, 5 mT).

## Applications

This work shows that even at low uniform magnetic fields, a non-magnetic island within a channel can be used to produce highly non-uniform distributions of magnetic and non-magnetic particles within the ferrofluid. This can be readily detected by changes in optical transparency in the vicinity of the island.

The magnetic forces applied on the ferrimagnetic particles, the “magnetic buoyancy” forces applied on the non-magnetic particles (e.g., bacteria) and the particle drag forces can be readily tuned by parameters such as particle size, magnetic properties etc. We have described a new method for applications such as detection and capture of magnetic and/or non-magnetic particles. Cell sorting using channels with appropriate geometric design, e.g., a sawtooth shaped channel side-boundary can also be envisaged. The optical transmission effect can be coupled with existing optical devices.

## Summary

An elliptical non-magnetic island in the middle of a channel was used, in the context of fluid flow in the channel, to experimentally and numerically study the distribution of magnetic nanoparticles and non-magnetic particles, such as bacteria in a carrier fluid. The island can be treated as a magnetic material with “relative-negative” susceptibility. The distortion of magnetic fields due to the island generates gradients of magnetic flux densities, resulting in magnetic forces. The magnetic particles accumulate near the island tips, while the non-magnetic particles (e.g., bacteria) accumulate near the center of the island. Each bacterium can concentrate the MNPs around its tips, and facilitate MNP alignment, even at low magnetic field. Interestingly, the interactions between bacteria and magnetic particles result in a net, with concentrated magnetic particles aligning along the magnetic field, cross linked and locked by the bacteria as nodes. Optical transmission was increased due to alignment of magnetic particles and by the presence of bacteria. This work would be useful for lab-on-a-chip applications such as detection and sorting of non-magnetic entities.

## Methodology

**Figure 5:**
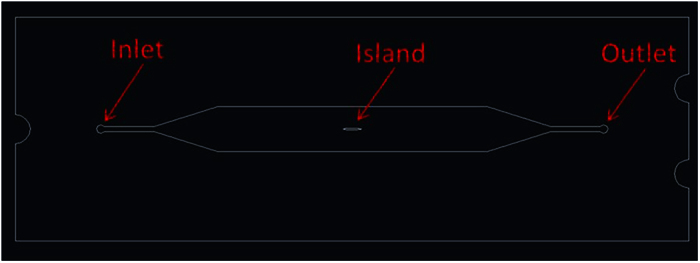
Shows a top view of the channel design in the chip, with an elliptical island in the middle.

Thermally bonded poly (methyl methacrylate) (PMMA) microfluidic chips were used for all experiments. A channel with one inlet and one outlet (Fig. 5) was fabricated by a standard computer numerical controlled (CNC) micro milling technique, followed by thermal bonding, by removing PMMA from the channel except a pillar of elliptical cross section (island) in the middle of the channel. This island, made of PMMA, had a semi-major axis of 1 mm and a semi-minor axis of 0.1 mm, with its major axis parallel to the chip direction (horizontal in Fig. 5). The rectangular middle testing section of the channel had a width of 5 mm and length of 30 mm. The depth of the channel, which was also the height of the island, was 47 ± 3 μm.

The chip was placed horizontally in a *uniform* magnetic field. The major axis direction of the elliptical island inside the channel was perpendicular or parallel (for fluid transparency tests only) to the magnetic field. A high speed camera (Phantom MIRO, M320S) was placed vertically above the chip to capture particle distribution inside the channel. A light source was placed below the chip with 30° shift from the vertical direction for most of the studies, except for the optical study where the light source was vertically placed below the chip. A syringe pump (KDS Gemini 88) and tubes were connected to the chip only to fill the fluids into the channel. The pump was switched off during testing and measurements, so that steady state studies of the ferrofluid were carried out with varying external *uniform* magnetic flux density. Preliminary tests were carried out to determine the useful range of bacteria concentration, MNP concentration and diameter and magnetic field strength.

## Materials

The black-brown color water based ferrofluid (EMG707, FerroTec) had a density (*ρ*_*ff*_) of 1100 kg/m^3^, dynamic viscosity (*η*_*ff*_) of 4.5 mPa.s, initial volume concentration (*C*_0_) of 2% and magnetic susceptibility *χ*_*ff*_ of 1.51 in SI units. The magnetic particles in the ferrofluid had an average diameter *d*_*mnp*_ = 2*r*_*mnp*_ = 10 nm. All the material properties of this kind of ferrofluid were offered by its supplier.

Bacillus Megaterium (Item # 154900A, CAROLINA) with an equivalent ball diameter of ~2.4 μm was prepared in water at a numerical concentration of 2.96 × 10^6^ cells/μl, which was confirmed by light microscope. The ferrofluid and bacteria mixture solution was prepared by mixing the ferrofluid with the Bacillus Megaterium-water solution, with a volume ratio of 2:1, so the final mixture has a bacteria numerical concentration of 9.87 × 10^5^ cells/μl. All the material properties of this kind of bacillus megaterium were offered by its supplier.

The chips and the islands were made of PMMA with a diamagnetic susceptibility of −2.10 × 10^−6^. Air has a paramagnetic susceptibility of 3.79 × 10^−7^. The bacillus megaterium has a susceptibility of 3.0 ~ 11.5 × 10^−16^, depending on its oxidation state and temperature^35^. Thus, the chip, island, air and bacteria have negligible magnetic susceptibilities.

## Numerical Simulation

The 2D modelling was performed using COMSOL Multiphysics (Extra fine mesh at the fluid and extremely fine mesh at the island, Fluid dynamics). The minimum and average quality was 0.7388 and 0.9786, respectively.

Equations 1 and 2 can be used to describe the magnetic fields:


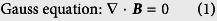



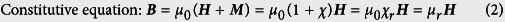


where *χ* and *χ*_*r*_ is the real susceptibility and relative susceptibility of the ferrofluid, respectively. The vectors ***B**, **H*** and ***M*** indicate the local magnetic flux density, magnetic field strength and magnetization, respectively. *μ*_0_ and *μ*_*r*_ represent vacuum permeability of free space and relative permeability of the material, respectively.

For the carrier fluids with non-zero magnetic susceptibility, the magnetic force (***F***_***m***_) applied on a particle with magnetic susceptibility of χ_p_ can be calculated as^24^,^36^


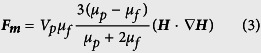


where *μ*_*p*_ = *μ*_0_(1 + *χ*_*p*_) and 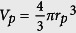 are the magnetic permeability and volume of a particle, respectively, and *μ*_*f*_ is the magnetic permeability of the carrier fluid. For MNPs and bacteria, the values of particle radius (*r*_*p*_) are 5 nm and 5 μm, respectively. The magnetic susceptibility of the particles (*χ*_*p*_) and bacteria are assumed to be *χ*_*ff*_ /*C*_0_ = 1.51/0.02 = 75.5 and 0, respectively.

The particle’s movement is governed by Newton’s second law:


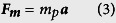


where *m*_*p*_ = *ρ*_*p*_*V*_*p*_ and vector ***a*** is the mass and the acceleration of the particle. The particle’s density (*ρ*_*p*_) 5170 and 1000 kg/m^3^ of MNPs and bacteria, are 5170 and 1000 kg/m^3^, respectively.

## Additional Information

**How to cite this article**: Wang, Z. M. *et al*. Magnetic Trapping of Bacteria at Low Magnetic Fields. *Sci. Rep.* **6**, 26945; doi: 10.1038/srep26945 (2016).

## Supplementary Material

Supplementary Information

Supplementary Information

## Figures and Tables

**Table 1:** Formulae relating real and converted susceptibilities of surrounding magnetic materials and island.

	**Real Values**		**Converted Values**		**Ratio of Relative Susceptibility**
	**Susceptibility,** ***χ***	**Relative Susceptibility,** ***χ***_***r***_** = *****χ***** + 1**	**Converted Susceptibility,** ***χ***_***con***_	**Converted Relative Susceptibility,** ***χ***_***con***_** + 1**	
Surrounding Material	*χ*_1_	1 + *χ*_1_	0	1	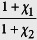
Island Material	*χ*_2_	1 + *χ*_2_	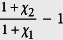	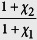	

## References

[b1] Rosensweig R. E. Magnetic Fluids. Annual Review of Fluid Mechanics 19, 437–461, doi: 10.1146/annurev.fl.19.010187.002253 (1987).

[b2] de Gans B. J., Blom C., Philipse A. P. & Mellema J. Linear viscoelasticity of an inverse ferrofluid. Physical Review E 60, 4518–4527 (1999). 10.1103/physreve.60.4518 11970308

[b3] Liang L. T., Zhu J. J. & Xuan X. C. Three-dimensional diamagnetic particle deflection in ferrofluid microchannel flows. Biomicrofluidics 5, 034110, doi: 10.1063/1.3618737 (2011). PMC3364825 22662037

[b4] Pamme N. & Manz A. On-Chip Free-Flow Magnetophoresis: Continuous Flow Separation of Magnetic Particles and Agglomerates. Analytical Chemistry 76, 7250–7256, doi: 10.1021/ac049183o (2004). 15595866

[b5] Ramadan Q. & Gijs M. M. Microfluidic applications of functionalized magnetic particles for environmental analysis: focus on waterborne pathogen detection. Microfluidics and Nanofluidics 13, 529–542, doi: 10.1007/s10404-012-1041-4 (2012).

[b6] Pamme N. On-chip bioanalysis with magnetic particles. Current Opinion in Chemical Biology 16, 436–443, doi: 10.1016/j.cbpa.2012.05.181 (2012). 22682892

[b7] Nguyen N.-T. Micro-magnetofluidics: interactions between magnetism and fluid flow on the microscale. Microfluidics and Nanofluidics 12, 1–16, doi: 10.1007/s10404-011-0903-5 (2012).

[b8] Suwa M. & Watarai H. Magnetoanalysis of micro/nanoparticles: A review. Analytica Chimica Acta 690, 137–147, doi: 10.1016/j.aca.2011.02.019 (2011). 21435469

[b9] Pamme N. Magnetism and microfluidics. Lab on a Chip 6, 24–38, doi: 10.1039/B513005K (2006). 16372066

[b10] Gijs M. M. Magnetic bead handling on-chip: new opportunities for analytical applications. Microfluidics and Nanofluidics 1, 22–40, doi: 10.1007/s10404-004-0010-y (2004).

[b11] Pamme N. & Wilhelm C. Continuous sorting of magnetic cells via on-chip free-flow magnetophoresis. Lab on a Chip 6, 974–980, doi: 10.1039/B604542A (2006). 16874365

[b12] Adams J. D., Thévoz P., Bruus H. & Soh H. T. Integrated acoustic and magnetic separation in microfluidic channels. Applied Physics Letters 95, 254103, doi: 10.1063/1.3275577 (2009). 20087428 PMC2807449

[b13] Plouffe B. D., Lewis L. H. & Murthy S. K. Computational design optimization for microfluidic magnetophoresis. Biomicrofluidics 5, 013413, doi: 10.1063/1.3553239 (2011). PMC3083238 21526007

[b14] Murariu V., Rotariu O., Badescu V. & Badescu R. Modelling of magnetodensimetric separation in two high gradient magnetic separation axial cells. Powder Technology 116, 97–102, doi: 10.1016/s0032-5910(00)00364-8 (2001).

[b15] Zeng J., Deng Y. X., Vedantam P., Tzeng T. R. & Xuan X. C. Magnetic separation of particles and cells in ferrofluid flow through a straight microchannel using two offset magnets. Journal of Magnetism and Magnetic Materials 346, 118–123, doi: 10.1016/j.jmmm.2013.07.021 (2013).

[b16] Zimmels Y., Tuval Y. & Lin I. J. Principles of high-gradient magnetogravimetric separation. Ieee Transactions on Magnetics 13, 1045–1052, doi: 10.1109/tmag.1977.1059516 (1977).

[b17] Rodriguez-Villarreal A. I. . Flow focussing of particles and cells based on their intrinsic properties using a simple diamagnetic repulsion setup. Lab on a Chip 11, 1240–1248, doi: 10.1039/C0LC00464B (2011). 21186390

[b18] Tarn M. D., Hirota N., Iles A. & Pamme N. On-chip diamagnetic repulsion in continuous flow. Science and Technology of Advanced Materials 10, 014611, doi: 10.1088/1468-6996/10/1/014611 (2009). 27877262 PMC5109609

[b19] Winkleman A. . A magnetic trap for living cells suspended in a paramagnetic buffer. Applied Physics Letters 85, 2411–2413, doi: 10.1063/1.1794372 (2004).

[b20] Wilbanks J. J. . Exploiting magnetic asymmetry to concentrate diamagnetic particles in ferrofluid microflows. Journal of Applied Physics 115, 044907, doi: 10.1063/1.4862965 (2014).

[b21] Song T., Pan H. M., Wang Z., Xiao T. & Wu L. F. Assessing bacterial magnetotactic behavior by using permanent magnet blocks. Chinese Science Bulletin 59, 1929–1935, doi: 10.1007/s11434-014-0298-2 (2014).

[b22] Fu L. M., Tsai C. H., Leong K. P. & Wen C. Y. Rapid Micromixer Via Ferrofluids. Physics Procedia 9, 270–273, doi: 10.1016/j.phpro.2010.11.060 (2010).

[b23] Furlani E. P. Analysis of particle transport in a magnetophoretic microsystem. Journal of Applied Physics 99, 024912, doi: 10.1063/1.2164531 (2006).

[b24] Furlani E. P. & Sahoo Y. Analytical model for the magnetic field and force in a magnetophoretic microsystem. Journal of Physics D-Applied Physics 39, 1724–1732, doi: 10.1088/0022-3727/39/9/003 (2006).

[b25] Furlani E. P., Sahoo Y., Ng K. C., Wortman J. C. & Monk T. E. A model for predicting magnetic particle capture in a microfluidic bioseparator. Biomedical Microdevices 9, 451–463, doi: 10.1007/s10544-007-9050-x (2007). 17516176

[b26] Skjeltorp A. T. One-dimensional and two-dimensional crystallization of magnetic holes. Physical Review Letters 51, 2306–2309, doi: 10.1103/PhysRevLett.51.2306 (1983).

[b27] Ido Y., Inagaki T. & Yamaguchi T. Numerical simulation of microstructure formation of suspended particles in magnetorheological fluids. Journal of Physics-Condensed Matter 22, 324103, doi: 10.1088/0953-8984/22/32/324103 (2010). 21386479

[b28] Patel R. Mechanism of chain formation in nanofluid based MR fluids. Journal of Magnetism and Magnetic Materials 323, 1360–1363, doi: 10.1016/j.jmmm.2010.11.046 (2011).

[b29] Zhu G. P. & Nguyen N. T. Magnetofluidic spreading in microchannels. Microfluidics and Nanofluidics 13, 655–663, doi: 10.1007/s10404-012-1056-x (2012).

[b30] Zhu G. P. & Nguyen N. T. Rapid magnetofluidic mixing in a uniform magnetic field. Lab on a Chip 12, 4772–4780, doi: 10.1039/c2lc40818j (2012). 22990170

[b31] McHenry M. E. . The Role of Surface Crystallography, Faceting and Chaining in Magnetic Applications of Nanoparticles and Nanocomposites. NEPTIS: Nisshin Engineering Particle Technology International Seminar 18, 5–14 (2009).

[b32] Collier K. N. . Controlled oxidation of FeCo magnetic nanoparticles to produce faceted FeCo/ferrite nanocomposites for rf heating applications. Journal of Applied Physics 105, 07A328, doi: 10.1063/1.3054376 (2009).

[b33] Wang Z., Varma V. B., Xia H. M., Wang Z. P. & Ramanujan R. V. Spreading of a ferrofluid core in three-stream micromixer channels. Physics of Fluids (1994-present) 27, 052004, doi: 10.1063/1.4919927 (2015).

[b34] Hanbury A. Constructing cylindrical coordinate colour spaces. Pattern Recognition Letters 29, 494–500, doi: 10.1016/j.patrec.2007.11.002 (2008).

[b35] Phillips W. D., McDonald C. C., Stombaug Na & Johnson W. H. O. Proton magnetic-resonance and magnetic-susceptibility characterization of ferredoxin-i from bacillus-polymyxa. Proceedings of the National Academy of Sciences of the United States of America 71, 140–143, doi: 10.1073/pnas.71.1.140 (1974). 4521046 PMC387952

[b36] Furlani E. P. Analysis of particle transport in a magnetophoretic microsystem. Journal of Applied Physics 99, doi: 10.1063/1.2164531 (2006).

