# Supplementary figures and images for: Magnetic Trapping of Bacteria at Low Magnetic Fields
Source: Sci Rep. 2016 Jun 2;6:26945. doi: 10.1038/srep26945 (PMC4890591; doi:10.1038/srep26945)

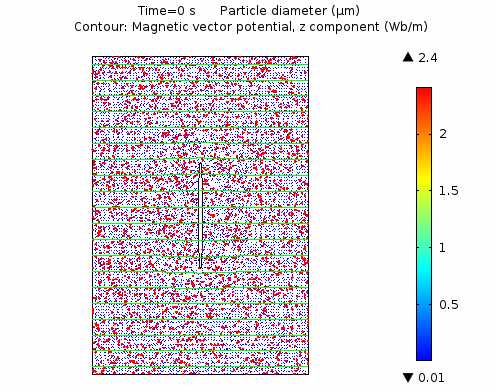

Supplement: Supplementary Information [file srep26945-s1.gif]
