# Supplementary material for: Magnetic Trapping of Bacteria at Low Magnetic Fields
Source: Sci Rep. 2016 Jun 2;6:26945. doi: 10.1038/srep26945 (PMC4890591; doi:10.1038/srep26945)
Supplement: Supplementary Information [file srep26945-s2.doc]

**Magnetic Trapping of Bacteria at Low Magnetic Fields**

Z. M. Wang^1^, R. G. Wu^2^, Z. P. Wang^2^, R. V. Ramanujan^1^*

^1^ School of Materials Science and Engineering, Nanyang Technological University

50 Nanyang Avenue, Singapore 639798, Singapore

^2^ Singapore Institute of Manufacturing Technology, 71 Nanyang Drive, Singapore 638075

**Video file legend**: S1

**Title of Video file**: MNPs and Bacteria Re-arrangements in Magnetic Fields

**Description of Video file**:

As shown in the video file (S1), the suspended non-magnetic particles (bacteria, red particles with bigger diameters of 2.4 µm) had “relative-negative” susceptibility compared with the ferrofluid, so the bacteria experienced negative magnetophoresis, and were repelled from the high magnetic field regions. One the other hand, the magnetic particles (MNPs, blue particles with smaller diameters of 0.1 µm) had “relative-positive” susceptibility compared with the ferrofluid, so the magnetic particles experienced positive magnetophoresis, and were attracted towards the high magnetic field regions.
